# Leveraging quality improvement through use of the Systems Assessment Tool in Indigenous primary health care services: a mixed methods study

**DOI:** 10.1186/s12913-016-1810-y

**Published:** 2016-10-18

**Authors:** Frances C. Cunningham, Sue Ferguson-Hill, Veronica Matthews, Ross Bailie

**Affiliations:** 1 Wellbeing and Preventable Chronic Disease Division, Menzies School of Health Research, Charles Darwin University, Spring Hill, Brisbane, Qld Australia; 2 National Centre for Quality Improvement in Indigenous Primary Health Care, Menzies School of Health Research, Charles Darwin University, Spring Hill, Brisbane, Qld Australia; 3 University Centre for Rural Health, University of Sydney, Sydney, Australia

**Keywords:** Chronic disease, Indigenous health, Primary health care, Quality improvement, Organisational assessment, Healthcare systems, Data collection tool

## Abstract

**Background:**

Assessment of the quality of primary health care health delivery systems is a vital part of continuous quality improvement (CQI) processes. The Systems Assessment Tool (SAT) was designed to support Indigenous PHC services in assessing and improving their health care systems. It was based on the Assessment of Chronic Illness Care scale, and on practical experience with applying systems assessments in quality improvement in Indigenous primary health care. We describe the development and application of the SAT, report on a survey to assess the utility of the SAT and review the use of the SAT in other CQI research programs.

**Methods:**

The mixed methods approach involved a review of documents and internal reports relating to experience with use of the SAT since its development in 2002 and a survey of key informants on their experience with using the SAT.

**Results:**

The paper drew from documents and internal reports to describe the SAT development and application in primary health care services from 2002 to 2014. Survey feedback highlighted the benefit to the whole primary health care team from participating in the SAT, bringing to light issues that might not emerge with separate individual tool completion. A majority of respondents reported changes in their health centres as a result of using the SAT. Good organisational and management support assisted with ensuring allocation of time and resources for SAT conduct. Respondents identified the importance of having a skilled, external facilitator.

**Conclusions:**

Originally designed as a measurement tool, the SAT rapidly evolved to become an important development tool, assisting teams in learning about primary health care system functioning, applying best practice and contributing to team strengthening. It is valued by primary health care centres as a lever in implementing improvements to strengthen centre delivery systems, and has potential for further adaptation and wider application in Australia and internationally.

**Electronic supplementary material:**

The online version of this article (doi:10.1186/s12913-016-1810-y) contains supplementary material, which is available to authorized users.

## Background

### Introduction

Australian Aboriginal and Torres Strait Islander (hereafter respectfully referred to as ‘Indigenous’) children born today can expect to live shorter lives than non-Indigenous children – 10.6 years shorter for males, and 9.5 years shorter for females [1]. Around two-thirds of the gap in health outcomes between Indigenous Australians and other Australians is due to long-term health problems [2]. Australian government policies highlight the need to address the gap in health status between Indigenous Australians and the general population [2, 3]. As it is a priority to improve Indigenous health status through the effective delivery of primary health care (PHC) services [4], there is a need for good quality tools to assess the effectiveness of the PHC systems providing care to Indigenous people.

A number of studies have identified the role of quality improvement (QI) methods in improving the effectiveness of health care delivery systems, and in health system strengthening [5, 6]. Chee et al. conceptualise health system strengthening as being “about permanently making the system function better, not just filling gaps or supporting the system to produce better short-term outcomes” [7]. This concept is fully supported in the World Health Organization (WHO) definition of health system strengthening: “improving [the] six health system building blocks and managing their interactions in ways that achieve more equitable and sustained improvements across health services and health outcomes” [5]. Providing evidence on the importance of addressing systems, a recent systematic review and meta-analysis of QI interventions for diabetes care in rural areas of Organisation for Economic Cooperation and Development countries found that successful QI interventions targeted both the health system and clinicians [8]. Nevertheless, a review by Rule et al. addressing the strengthening of PHC in low- and middle-income countries found no consistent approach for assessing the effectiveness of PHC delivery in those countries [9].

The delivery of PHC for Indigenous people in Australia is provided through three main service providers: Indigenous community-controlled health services, state and territory government funded and/or operated services, and general practitioners in private practice [10]. The Indigenous community controlled health service sector provides PHC through community health centres operating under a variety of funding models [11]. These services operate in the metropolitan, regional, rural and remote areas of all states and territories in Australia, and are funded primarily through national Australian government funding. Indigenous controlled services are controlled by, and accountable to, Indigenous people in those areas in which they operate. The services aim to provide culturally appropriate health care to the community that controls the service. State and territory government funded and/or operated services also provide Indigenous PHC services, predominantly where there have been gaps in existing PHC service provision. In addition, access to PHC services for Indigenous Australians may be through the mainstream services of general practitioners in private practice, funded through Medicare, the national universal health insurance program.

The Systems Assessment Tool (SAT) was developed to assess the organisational systems of such diverse health care services supporting Indigenous PHC. This paper describes the development and application of the SAT, it assesses the utility of the tool based on the results of a 2014 survey of key informants involved in using the SAT in their health centres as part of their continuous quality improvement (CQI) processes, and it reviews the wider use of the SAT in other CQI research programs.

### Development of the SAT scale

The SAT is an Australian developed scale, based on the Assessment of Chronic Illness Care (ACIC, Version 3.5) scale [12], for application in Indigenous PHC settings. The ACIC scale is a validated 28-item measure of the extent to which care delivered is consistent with the Chronic Care Model [13–15]. The Chronic Care Model describes characteristics of a clinic that, if present, should result in improved chronic illness care – this model has been successfully implemented in a wide range of healthcare organisations [16]. Bonomi et al. [13] reported on the evaluation of the ACIC tool, demonstrating its sensitivity to detecting system improvements in two chronic illness care areas: diabetes and congestive heart failure. Of the tools designed to measure organisational attributes associated with effective chronic disease management in PHC settings, the ACIC tool is most cited in publications [17]. Research by Parchman et al. [18] confirmed that the characteristics of primary care clinics delivering care to type 2 diabetes patients (as measured by the clinics’ ACIC scores) were an important predicator of glucose control.

In 2002, researchers from the Menzies School of Health Research used a participatory action research approach [19] in the Audit and Best Practice in Chronic Disease (ABCD) Project to modify the ACIC scale for the development of the SAT (with permission from the MacColl Institute for Healthcare Innovation) [20]. While retaining the basic structure and dimensions of the ACIC scale, the modifications were to facilitate use of the scale in assessing chronic health care delivery for CQI, as part of the ‘Plan-Do-Study-Act’ QI cycle in Indigenous PHC settings in Australia. It had to be possible to use the scale in conjunction with review of clinical audit data of scheduled services provided in different areas of health care. These requirements necessitated substantial development work on descriptors for the scoring, and on refining the wording for local use. The structure of the SAT also drew on the WHO Innovative Care for Chronic Conditions (ICCC) Framework [21]. Unlike the ACIC scale, the SAT was developed to be delivered in a group setting to members of a health centre team by an experienced and trained facilitator.

The SAT and its use in assessing the status of health centre systems for chronic illness care was described in a 2005 study of the ABCD CQI project undertaken in 12 Aboriginal community health centres in the Top End of the Northern Territory, Australia [20, 22, 23]. In an application of linear regression modelling in the ABCD study of 12 Aboriginal community health centres, Si et al. [20] found that each systems assessment component was statistically associated with overall adherence to delivery of type 2 diabetes services. Si et al. concluded that the SAT should be useful in assessing and guiding development of systems for improvement in diabetes care in similar settings in Australia and internationally. The ABCD study findings on systems improvement, compared with baseline data, were reported by Bailie et al. [11] to be very similar to those found in the first of the “Breakthrough” series in the US [24].

From its initial 2002-2005 application in 12 Northern Territory health centres in the ABCD project, use of the SAT spread from 2005-2009 through an extension phase of the ABCD project to 63 health centres in four Australian states and territories [25]. Twelve of the 14 centres which had completed the systems assessment at baseline and at the end of two subsequent CQI cycles had clear evidence of improvement in the health centre systems required to support best practice care [26]. A number of those participating services reported that they used the approach to support a system-based change management process for reorienting PHC from an episodic acute care model to a chronic care model [26]. The ABCD National Research Partnership extended the ABCD program of work from 2010 to 2015 [27].

The SAT is applicable to Indigenous and non-Indigenous PHC settings, and it has been applied in both settings. Following the original application of the SAT to the management of health services provided for chronic disease systems, the tool was adapted for use in preventive health, maternal health, child health, rheumatic heart disease, mental health, and health promotion service provision. The current SAT is a generic tool which builds on service-level experience, that can be used for any client group, with potential for inclusion of some condition specific items. However, there is a specific health promotion SAT (described in more detail below).

The tool is supported through the online system of a not-for-profit service agency, One21seventy, at Menzies School of Health Research. One21seventy develops and maintains evidence-based audit and systems assessment tools, provides online data services for automated reporting, benchmarking and interpretation, and training and site support for conducting audits according to standard protocols. One21seventy clinical audit tools provide data on the following clinical areas: preventive health, child health, maternal health, mental health, rheumatic heart disease, sexual health, vascular and metabolic syndrome management, youth health, and health promotion services. The ABCD National Research Partnership worked alongside One21seventy from 2010 to 2015 to develop the evidence base in continuous quality improvement in PHC.

### SAT elements and item scoring

The tool provides for (1) a structured assessment of the enablers and barriers in health centre systems that support good clinical care; (2) guidance on the next steps in planning improvements; and (3) assessment of progress in achieving system improvement. The SAT has five components, with a total of 20 items (Table 1): (1) *delivery system design* (8 items), (2) *information systems and decision support* (3 items), (3) *self-management support* (2 items), (4) *links with community, other health services and other services* (4 items), and (5) *organisational influence and integration* (3 items).

**Table 1:** Systems assessment tool components

Components of systems	Items for each component
1. Delivery system design (eight items)	1. Team structure and function
	2. Clinical leadership
	3. Appointments and scheduling
	4. Care planning
	5. Systematic approach to follow-up
	6. Continuity of care
	7. Client access/cultural competence
	8. Physical infrastructure, supplies and equipment
2. Information systems and decision support (three items)	1. Maintenance and use of electronic client lists
	2. Evidence-based guidelines
	3. Specialist-generalist collaborations
3. Self-management support (two items)	1. Assessment and documentation
	2. Self-management education and support, behavioural risk reduction and peer support
4. Links with the community, other health services and other services (four items)	1. Communication and cooperation on governance and operation of the health centre and other community based organisations and programs
	2. Linking health centre clients to outside resources
	3. Working in the community
	4. Communication and cooperation on regional health planning and development of health resources
5. Organisational influence and integration (three items)	1. Organisational commitment
	2. Quality improvement strategies
	3. Integration of health system components

The assessment for each item in the scale requires recording of a score in the range of 0-11: the higher the score, the better the items are ranked. The scale includes a detailed set of prompts to increase standardisation and reproducibility in scoring. Health centre staff are requested to provide a qualitative justification for their score in relation to these prompts. The scores are categorised as: 0-2 (limited support); 3-5 (basic support); 6-8 (good support); and 9-11 (fully developed support). The score and justification for each item is obtained by arriving at a consensus among participating health centre staff members. The mean is calculated from individual item scores to create a component score, and the mean of five component scores forms the overall system score for the centre. The SAT requires different scores and justifications to be made for each clinical audit tool (chronic disease, maternal health, etc.). This is because the assessment needs to address the quality of delivery systems supporting the different clinical care areas.

### Conduct of the SAT

The SAT is intended to be delivered with facilitated discussion as part of step 3 of the 5 step structured plan-do-study-act CQI cycle of One21seventy (Step 1. Signed agreement; Step 2, Training/Orientation; Step 3. Audits, system assessment; Step 4. Participatory interpretation,

data analysis and report preparation; Step 5. Action planning; Step 6. Act, implement changes. Then, back to Step 3 …). Health centre staff conduct their clinical audits in Step 3, and they are also encouraged to conduct the SAT in this step of their CQI cycle. Health services are thus able to use the SAT to assess their systems for the delivery of care, and the SAT data and the clinical audit data are used together as a basis for planning action and implementing change. However, it should be noted that as the SAT is available on an open-access basis, it may also be used as a tool for direct assessment of a service’s organisational systems independent of the One21seventy CQI cycle and the One21seventy suite of tools.

**Fig. 1:**
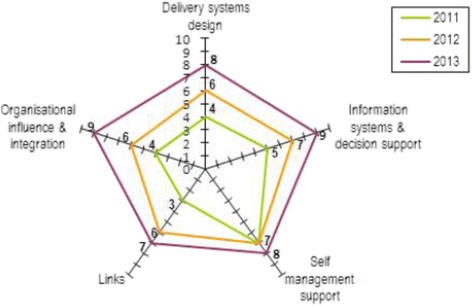
Radar plot, displaying annual SAT component scores for one health centre in 2011, 2012 and 2013

The Systems Assessment Tool (Additional file 1) is supported by a Facilitator’s Guide (Additional file 2) [28], and a SAT scoring form (Additional file 3). Help-desk assistance is available through One21seventy. The systems assessment scores and justifications are entered into the One21seventy web-based information system and these data are used to generate downloadable reports for the health centre, including ‘radar plots’ of the service’s systems assessment, displaying the relative strengths and weaknesses of system components for the health centre. An example of a ‘radar plot’ diagram showing SAT results for one health centre for the five components at 2010 (baseline), 2012 and 2013 is provided in Fig. 1. SAT improvements in scores over time are reflected on the axes. The data analysis and reporting also includes the capacity for centres to compare their performance data with others in their region.

## Methods

**Fig. 2:**
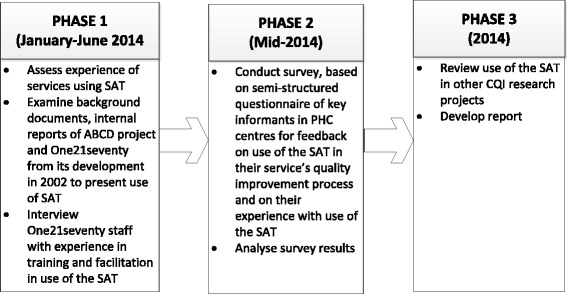
Study Phases

A 3-phase, mixed-methods approach was applied to assess the utility of the SAT (Fig. 2). Phase 1 included assessment of the experience of services with using the SAT through examination of background documents and internal reports of the ABCD project and One21seventy relating to the development and experience of Indigenous PHC centres with using the SAT from 2002 to 2014.

Interviews were conducted with three One21seventy staff with experience in training and facilitation in the use of the SAT. In Phase 2, a semi-structured questionnaire (Table 2) was used to conduct a survey in mid-2014 of 25 key informants in PHC centres to elicit their feedback on how they have used the SAT as part of their service’s quality improvement process, and on their experience with use of the SAT. In Phase 3 a review was undertaken of the use of the SAT in CQI research programs, based on advice provided in the interviews with One21seventy staff and from the first-hand knowledge of the authors of this paper, as well as through the retrieval of published research articles referring to use of the SAT in their study methods.

**Table 2:** Feedback survey on systems assessment tool (SAT)

**ᅟ**
**1. Date of completion**: / / / **2. Your name (**optional**): ______________________________________________** **3. What is your role/position:** __________________________________________ **4. Name of your health service** ***(or area or region that you look after)*** **:** _______________________________________ a. **Location of health service (** ***please circle*** **):** Urban/Regional/Remote **5. Has your health service (or any of the services that you facilitate) used the SAT in the last 12 months? Yes [ ]– go to Q8. No [ ]– go to Q6** (*please place* ***X*** *in the appropriate box here and for questions below*) **6. Has your health service (or services that you look after) previously used the SAT? Yes [ ] – go to Q7. No [ ] – go to Q10 (Could you please comment in Q10 on why the SAT has not been used?)** **7. How long ago was the last use of SAT in your service (or service that you facilitate?)** **Service 1 [--.—years ago]** **Service 2 [--.—years ago]** **Service 3 [--.—years ago]** **8. Thinking of the most recent use of the SAT tool you were involved in:** **a. Approximately how many service people participated in the SAT session? ___** **b. Who participated in the assessment?** (1) nurse [Yes__][No__] (2) Aboriginal and/or Torres Strait Islander Health Worker [Yes__] [No__] (3) administrative staff [Yes__] [No__] (4) management staff [Yes__] [No__] (5) general practitioner [Yes__] [No__] (6) allied health staff [Yes__] [No__] (7) driver [Yes__] [No__] (8) community representatives [Yes__] [No__] (9) service clients [Yes__] [No__] (10) staff from other organisations [Yes__] [No__] (11) other.[Yes__][No__] If yes, what was their role/ position?: ___________________________________________ c. **Did the health service benefit from using the SAT? Yes []: please explain what the benefits were: If No []: - go to 8d:** (1)_______________________________________________________________________________________________________ (2)____________________________________________________________________________________ **d. Were any problems or difficulties experienced with the last use of SAT?** (1)____________________________________________________________________________________ (2)____________________________________________________________________________________ **e. Did the health service make any practice changes as a result of findings from the SAT?** (1) Yes [ ] Go to **Q8f** (2) No [ ] – Do you know why not? ____________________________________________________________________________ **f. What changes did the health service make?** _______________________________________________________________________ **9. Please provide feedback on the different ways the SAT tool is being delivered or facilitated:** _____________________________________________________________________________________________________________________ _________________________________________________________________________________________________ **10. Do you have any other comments?** _____________________________________________________________________________________________________________________

Phase 2 of the study used a purposive sample of 25 informants in Indigenous PHC centres participating in the ABCD Partnership. Of the 17 respondents (68 % response rate) to the survey (who consented to their participation in the survey by return email), the majority of the survey responses (15) were obtained through telephone interviews (conducted by FC), with two respondents returning their completed surveys by email. The respondents to the mid-2014 survey represented Indigenous PHC centres and providers from four jurisdictions, Northern Territory, Queensland, New South Wales and South Australia, and represented centres in urban, regional and remote settings, and community controlled health centres and government operated centres. Respondents included CQI facilitators (10) (a number of whom have used the SAT extensively across numerous health centres over successive years), health service managers (4), clinical directors (1) and advisers (2).

Survey responses were de-identified and entered into a database to facilitate comparative and thematic analysis. The thematic analysis was based on a thorough, inclusive and comprehensive review of the survey data. All relevant extracts for each theme were collated. Themes were checked against each other and back to the original data set, and further checked to ensure their coherence and consistency. To assist with interpretation of results, initial study findings were presented and discussed with participants in the October 2014 ABCD National Research Partnership biannual meeting. The majority of these participants represented or were affiliated with services that had used the One21seventy tools including the SAT.

## Results

### Document review

Materials, internal reports, SAT guides and published articles dating from the initial development of the SAT in 2002 to the end of 2014 were reviewed to inform this study. The material presented in the earlier sections of this paper on the development of the SAT scale, the description of the SAT elements and item scoring, and the conduct of the SAT has been based on a comprehensive review of this material.

### Analysis of feedback on use of the SAT

The mid-2014 survey provided feedback from PHC centres on their experience with using the SAT. Respondents reported that the number of participants involved in the conduct of the systems assessment at their centre generally ranged from four to 22 (mean: 6). These health centre participants included centre nurses, Aboriginal and Torres Strait Islander Health Workers, administrative staff, management, general practitioners (participating by tele- or video-conference in some remote settings), and allied health practitioners. A number of centres also included their patient transport driver, and a representative from the state or territory level Aboriginal Controlled Health Organisation. One service included a community representative.

The following section provides findings from the thematic analysis of survey responses. The survey questions asked respondents to comment on benefits (if any) to their centre from use of the SAT, on any issues or difficulties experienced with use of the SAT, on whether the centre made any practice changes as a result of findings from the SAT, and if so, what these changes were, and to provide feedback on the different ways that the tool is being delivered or facilitated. Representative respondent comments reflecting key themes are set out in Table 3.

**Table 3:** Respondent feedback on experience with using the SAT

Theme	Representative respondent comments
Benefit to health centre from using SAT	[The SAT allowed the centre to] *“reflect on systems and system utilisation, identify differences between programs in system utilisation, and to identify barriers/issues in systems.* (respondent02) *“Many clinic managers conveyed that they felt more in control of all the various challenges and could see the linkages and a map to help move forward.”* [respondent11) [The SAT] *“supported clinic staff to discuss challenges within a safe space with management [to] help guide immediate/future planning.”* (respondent11) *“Focussed, directed planning time.”* (respondent13) *“Reflect on clinical practices – measure gaps and achievements; reflect on processes, documentation and services delivery.”* (respondent14) *“Facilitated group discussion and decision-making; enabled staff to identify the things that they were doing well.”* (respondent15)
Benefit to team from using SAT	*“Team together, and talking.”* (respondent06) *“Great opportunity for team building.”* (respondent07) *“Developing, expressing ideas. Coming together as a team – much needed – brilliant.”* (respondent08) *“Team building.”* (respondent10) *“Shared understanding of role within the service. Focused, directed planning time.”* (respondent13) *“Audit process and SAT was a very valuable experience for the whole team. All staff enjoyed and benefited from auditing/audit results and SAT – identifying issues and gaps and then planning to improve our service by adapting.”* (respondent14)
Different applications of SAT in health centres	[The regional service has always] *“done interpretation, feedback, the SAT, and goal setting in one session.”* (respondent13)
Changes in health centres resulting from conduct of SAT	*“Recommended the manager identify under-utilisation in services, and incorporate this in the Action Plan.”* (respondent02) *“Placed prompts in prominent places.”* (respondent04) *“Team became cohesive, with changes in staff/time.”* (respondent08) *“Change in role of health promotion in organisation. Increased forms of health promotion in the organisation and this happened by the appointment of a new staff member.”* (respondent10) *“Tried to police what was really affecting them. Most wanted more staff – couldn’t address.”* (respondent12) [Addressed] “*documentation”* (respondent14) *“Especially when they ran the SAT two times – improved processes. They went through a time of change and their results picked up.” (*respondent16) *“The information that is gathered in the SAT was used as evidence to make recommendations for improvements in the PHC system. Things like org* [organisational] *structure, policies and procedures and health promotion.” “Many changes … structural and process changes were made as a result of the SAT enquiry.”* (respondent 17).
Issues relating to SAT use and facilitation	*“Time required. Difficulty maintaining staff interest”* (respondent03) “*Resulted in some disagreement and debate. Managers (or other positions of authority/power) tended to have final say.”* (respondent04) “[the services] *use an external facilitator for the SAT process. This makes it easier for staff within the organisation to participate openly in the process. Expert facilitators who have a good understanding of the service delivery context should be used when implementing the SAT. It is often the case that there are issues within a health service or between people within the health service that will be aired during the SAT. This can be dangerous if the facilitator does not know to expect this and have the necessary skills to divert the discussion. Also there will generally be someone in the discussion who dominates and the facilitator needs to be able to balance the impact of the dominant voice …”* (respondent17)

#### Theme 1: benefits to health centres from SAT use

Feedback was provided on respondent perceptions of benefits (if any) to their health centre from use of the SAT. Respondents generally provided positive feedback with examples of benefits to their centres from using the SAT. For example, the SAT facilitated group discussion and decision-making: the SAT *‘focused, directed planning time’* (respondent13). The process encouraged centres to reflect on their clinical practices, processes, documentation and service delivery, measuring gaps and achievements. One respondent commented: *“Supported clinic staff to discuss challenges within [a] safe space with management. Help[ed] guide immediate/future planning … Many clinic managers conveyed that they felt more in control of all the various challenges and could see the linkages and a map to help move forward*” (respondent11). Use of the SAT increased participants’ understanding of population health approaches and the Chronic Care Model.

#### Theme 2: whole team participation in systems assessment

A common theme from respondents reflected the benefits to the whole team from participating in the systems assessment, in terms of team building and establishing shared understandings. Feedback highlighted the importance of the SAT dialogue in helping teams to understand what a system is, and in formulating a common view of the weaknesses and strengths of health centre systems in relation to best practice. In addition, respondents identified the importance of the no-blame approach that underpins the philosophy of a systems approach in addressing the weaknesses and strengths in service delivery. All members of the PHC centre are invited to be present when conducting the SAT – health centre staff and managers (including their clinicians plus visiting clinicians, managers, receptionists, patient transport drivers and cleaners). This may be the only time that this occurs on a whole-of-health-centre basis. A group of people who have insight into the centre systems issues is brought together, and a discussion is facilitated around the components of the SAT. Completion of the SAT through group discussion of the issues brings to light issues that may not emerge if the SAT is completed separately by each individual. A number of services quarantine time and bring in relief staff to backfill positions in the clinic so that the entire team can attend. As conveyed by one respondent : “*Audit process and SAT was a very valuable experience for the whole team. All staff enjoyed and benefitted from auditing/audit results and SAT – identifying issues and gaps and then planning to improve our service by adapting”* (respondent14).

#### Theme 3: changes made to health centres

Respondents were asked to comment on whether there had been changes in their health centre as a result of the conduct of the SAT. Twelve of the 17 respondents said that their centre had introduced changes. These changes ranged from taking action to address under-utilisation in services, incorporating prompts in their clinical information systems, improving documentation, improving policies and procedures, and changing the centre’s organisational structure.

#### Theme 4: different approaches to using the SAT

There was respondent comment on the different ways that the SAT has been used in their health centres. For example, in some South Australian health centres, the tool was facilitated by in-house managers to their team during the weekly staff meeting, while other centres used funding available for accreditation to hire external facilitators to conduct the systems assessment. One respondent commented that, to keep momentum and enthusiasm at a high level, their regional service has always *“done interpretation, feedback, the SAT, and goal setting in one session”* (respondent13). The conduct of the SAT can be very flexible. Some health centres conduct a generic systems assessment, while others may do a number of separate systems assessments, using the SAT and addressing the specific clinical audit care areas. As part of their CQI process using the One21seventy CQI cycle, health centres can examine their audit data reports, and in the goal setting phase, the centre can choose what they should focus on based on their data. Some centres conduct the SAT on an annual basis, while others do not believe it needs to be conducted each year as their systems have not changed.

#### Theme 5: challenges in using SAT

A number of the respondents commented on the challenge for the health centre in finding the time to get all the staff and service representatives together to complete the tool, because there is a lack of allocated time for specific CQI processes (ie, time not committed to direct service delivery) available in practice settings. This requires resourcing, protected time and commitment from management, and there can be challenges in securing organisational back-up, particularly in remote areas. The time required to conduct the SAT was reported as an issue by some respondents, for example one respondent noted: *“Lengthy process not unusual [for this type of assessment], but noted”* (respondent13)*.*

#### Theme 6: role of facilitator

The importance of having a skilled facilitator was highlighted in responses. For example, one respondent’s comments reflected the situation where there was a lack of a skilled facilitator: *“Dominant personalities influenced the scoring, less outspoken participants had tendency to give-in to compromise of manager’s opinion”* (respondent04). A number of respondents commented on the need to have an external facilitator – experienced, external facilitators who understand the purpose and content of the SAT help to focus the discussion on the important issues for systems assessment. A good facilitator helps to ensure that all members of the group are able to participate and share their views. *“This makes it easier for staff within the organisation to participate openly in the process”* (respondent17). As different staff may have very different perspectives on how the systems function, this contributes to a wider shared understanding of the strengths and weaknesses in the system. A facilitator can also help tease out areas where there is good consensus and areas where there are different perspectives regarding how well systems are working.

### Review of SAT use in CQI research projects

There are a number of examples showing how the SAT has been used in research studies as an objective measure of the ‘strength’ of health service systems. The ABCD National Research Partnership worked with health service staff, management and policy makers in five Australian jurisdictions to enhance the effective implementation of successful strategies to improve Indigenous primary care performance [27]. Data from application of the SAT tool by Partnership participating centres is being used, in addition to their clinical audit data, in analysis of variation between centres in the quality of care and intermediate health outcomes. As reported in the final report on Chronic Illness Care for Aboriginal and Torres Islander people from the Engaging Stakeholders in Identifying Priority Evidence-Practice Gaps and Strategies for Improvement in Primary Health Care Project [29], analysis of health service SAT data highlighted the need to strengthen systems for more effective links between health centres and communities, and links with other health services and resources.

The SAT has been used in a number of additional CQI applications. One recent application is in CQI in health promotion for PHC services. As health promotion is integral to comprehensive PHC, CQI in health promotion is also integral to quality improvement across a range of PHC services. A suite of health promotion CQI tools were recently developed by Percival [30] as an innovative approach for assessing PHC-based health promotion activities, based on the CQI technique of audit and feedback cycles. The tools are available through One21seventy. An adaptation of the SAT was included as a component of these tools. The CQI tools and supporting resources were developed for use by multidisciplinary teams with varying levels of health promotion expertise. The systems assessment component enables a service to assess how well organisational systems are functioning to support health promotion in (1) the service delivery system, (2) information systems and decision support, (3) adaptability and integration of systems and (4) the organisational environment. O’Donoghue et al. [31] reported on the acceptability and feasibility of use of these tools in four Indigenous PHC services in the Northern Territory, Australia. Facilitated participatory processes were found to be important for the collection of locally relevant information and for contributing to improving PHC practitioners’ knowledge and understanding of best practice health promotion.

Peiris et al. [32] employed an adapted SAT as part of the Kanyini Vascular Collaboration study, established in 2006 with a focus on vascular diseases. The tool helped with exploring Aboriginal Medical Service staff views on factors needed to improve chronic care systems. Four principal domains of inquiry were employed in the tool to suit the local context: (1) health service governance and cultural safety; (2) workforce issues and professional standards; (3) experiences of QI activities and supports; and (4) navigation of care including access to hospital and specialist services. In the STRIVE study (Sexually Transmitted Infections in Remote Communities: Improved and Enhanced PHC), an adaptation of the SAT was used to provide an annual assessment of the current status of sexual health service delivery at study services [33].

Researchers from Menzies School of Health Research are collaborating with Canadian researchers at Western University, Ontario in employing an adaptation of the SAT in a five-year (2013-2017) study, Transformation of Indigenous Primary Healthcare Delivery (FORGE AHEAD). The study is aimed at developing community-driven, primary healthcare models that enhance chronic disease management, particularly of type 2 diabetes, in First Nations communities across Canada [34]. An adaptation of the SAT is being used to collect baseline and trend data to assess current PHC delivery systems in the First Nations communities.

## Discussion

This paper provides background on the development of the SAT to assist Indigenous PHC services in assessing the performance of their health care systems. The tool helps teams focus their efforts on adopting evidence-based changes to improve practice. The standardised tool allows assessments to be conducted in a comparable way at different times and in different places, in a way that covers the key components of health centre organisational systems [35]. If health centres employ their own, individually developed, system appraisals as suggested in one paper [36], such essential features are likely to be lost. Centres need resourcing, protected time and commitment from management to undertake the SAT to improve the quality of systems assessments. The study findings regarding the important role of skilled facilitators, and the need for protected time, in the SAT process are consistent with the model of facilitation developed by Rhydderch et al. to improve organisational development in UK primary care practices [37]. The identification of the crucial role of the facilitator in the SAT process supports the findings of Parchman et al. from a US randomised trial of practice facilitation to improve the delivery of chronic illness care in primary care [38]. Practice facilitation resulted in a significant and sustained improvement in delivery of care consistent with the Chronic Care Model as reported by those involved in direct patient care in small primary care practices. Similarly, when the SAT was initially used in the ABCD study there were hub coordinators or other people who coordinated the SAT in PHC centres providing services to Indigenous people in the Northern Territory, Queensland and Far West New South Wales. Following the research phase of the ABCD study, the SAT was coordinated through CQI facilitators in the Northern Territory, Queensland, Western Australia and South Australia.

The information on the use of the SAT in this study is based on interviews with key informants working within One21seventy who have first-hand knowledge and experience with use of the SAT, on feedback from 17 health centre respondents to a survey, and on a review of the wider use of the SAT in CQI research projects. Although survey respondents represented the range of jurisdictions where the SAT has been employed, there may be limitations to the generalizability of study findings as there may be variation in the process of conducting the SAT, and the use of the SAT data, by individual PHC centres.

## Conclusions

The SAT was originally designed as a measurement tool, however it rapidly evolved to become an important developmental tool, enabling team learning about PHC system functioning in relation to articulated best practice, and contributing to team strengthening. The tool provides a framework for health centre teams to discuss components related to effective health care in PHC settings, and score justifications, leading to a better understanding among staff of the quality of their centre’s systems and to their identification of priorities for systems improvement, and monitoring and evaluation of progress towards their goals [11]. It is critical to have both local health centre management support and trained facilitators for successful conduct of the SAT. The SAT has demonstrated its importance as a component of the CQI cycle, based on clinical audit and SAT feedback for action planning and implementation. As the systems assessment requires local health centre staff and managers to discuss and come to a consensus about how well their systems are working, use of the tool can – in itself – serve as an important change process for the health service.

There is substantial Australian experience with the effective practical application of the SAT in conjunction with data from clinical audit tools in assisting Indigenous PHC centres with their CQI processes. The SAT has also been applied in a range of other quality improvement research projects in Australia and internationally. Based on this experience, there is potential for the SAT to be employed more widely in other PHC settings in Australia and internationally.

## Additional files

Additional file 1: Systems Assessment Tool – All client groups, Version 2.0. (PDF 218 kb)
Additional file 2: Facilitator’s Guide to Systems Assessment Tool, Version 1.0. (PDF 421 kb)
Additional file 3: Systems Assessment Scoring Form, Version 2.0. (PDF 74 kb)

## Abbreviations

ABCD: Audit and best practice for chronic disease program
ACIC: Assessment of Chronic Illness Care
CQI: Continuous quality improvement
PHC: Primary health care
QI: Quality improvement
SAT: Systems assessment tool
UK: United Kingdom
US: United States
WHO: World Health Organisation.
