# Supplementary material for: Leveraging quality improvement through use of the Systems Assessment Tool in Indigenous primary health care services: a mixed methods study
Source: BMC Health Serv Res. 2016 Oct 18;16:583. doi: 10.1186/s12913-016-1810-y (PMC5070177; doi:10.1186/s12913-016-1810-y)
Supplement: Additional file 1: — Systems Assessment Tool – All client groups, Version 2.0. (PDF 218 kb) [file 12913_2016_1810_MOESM1_ESM.pdf]

Systems assessment tool – All client groups

Version 2.0

September 2012

One21seventy
National Centre for Quality Improvement in Indigenous Primary Health Care

menzies school of health research

the Lowitja Institute
Australia's National Institute for Aboriginal and Torres Strait Islander Health Research

Incorporating the Cooperative Research Centre for Aboriginal and Torres Strait Islander Health

© Menzies School of Health Research 2012

This material is copyright. Apart from use permitted under the *Copyright Act 1968* (Cwlth), all other rights are reserved. No part of this material may be reproduced, by any process, without prior written permission. Parts of this material are available to registered users for download from the One21seventy web-based information system, www.one21seventy.org.au. Requests and enquiries concerning reproduction and rights should be addressed to: Menzies School of Health Research, Darwin Northern Territory, Australia.

First published 2009

Acknowledgments

We would like to acknowledge and thank all the members who participated in the working group:

Jenny Brands, Michel Burgum, Kerry Copley, Cynthia Croft, Sue Ferguson-Hill, Jenny Hains, Cath Kennedy, Ru Kwedza, Alison Laycock, Diana Mosca, Estrella Munoz, Louise Patel, Letitia Robinson.

Contents

A tool for assessment of health centre systems to support primary health care 1

Components of the Systems Assessment 3

Component 1 Delivery system design 5

Component 2 Information systems and decision support 13

Component 3 Self-management support 16

Component 4 Links with community, other health services and resources 18

Component 5 Organisational influence and integration 23

Version control

Version	Release date	Description
1.2	18 November 2010	Release
2.0	21 September 2012	Release

A tool for assessment of health centre systems to support primary health care

It is widely recognised that primary health care organisations need to be re-oriented to more effectively address the health challenges of the 21st century. Chronic conditions are responsible for a large and increasing burden of illness in communities and make up an increasingly important part of the workload of primary health care centres. Health during pregnancy and early childhood has long term and wide ranging impacts on health, including on the incidence of chronic disease. Mental health concerns are imposing an increasingly heavy burden on the community and on primary health care services. Promotion of good health in general and for priority groups such as pregnant women and children is recognised as an important function for primary health care organisations.

In the context of demands for episodic acute care, systems need to be put in place to meet the ongoing needs of specific client groups. Health centres need practical tools to guide these efforts and to evaluate changes made to their service delivery systems. The Systems Assessment Tool (SAT) has been designed for use by organisations providing primary health care services for Indigenous Australian populations. However, it is expected to be appropriate with minor adaptation for many other settings.

The SAT has evolved from the Chronic Care Model and the associated Assessment of Chronic Illness Care (ACIC) tool (Bonomi et al., 2002) and from the Innovative Care for Chronic Conditions (ICCC) Framework (WHO 2002). It was originally designed for assessing systems for chronic disease care, then adapted for use for maternal and child health.

This Generic SAT builds on our experience of using these earlier specific tools, to provide a single tool that can be used for any client group.

The intended purpose of the tool is to support ongoing quality improvement initiatives through systematic assessment of a range of elements of health centre systems that have been demonstrated to be important. The tool provides for:

- an assessment of the state of development of health centre systems;
- guidance on next steps in planning improvements; and
- assessment of progress in achieving system improvement.

The SAT incorporates the guiding principles of the ICCC Framework: evidence-based decision making; population focus; prevention focus; quality focus; integration; and flexibility/adaptability.

Services are of three types:

1. *Client clinical care services* for those with a diagnosed disease or condition (including pregnancy) – generally health centre based, one-to-one activities
2. *Client services for the prevention and early detection of disease* (including screening, growth monitoring, case finding, brief interventions/counselling – generally health centre based, one-to-one activities but may also include group activities
3. *Population programs and activities* (eg to promote nutrition, breastfeeding, physical activity, oral/dental health, mental health, environmental health, and to reduce harm from tobacco smoke or alcohol) – generally community based

Each of these three types of services is important in effective primary health care. The quality of systems in place to support them may differ quite markedly within the same health centre, both within and between client groups.

The prompts provided in the tool are intended only as a guide to some of the sorts of system issues that one might consider for scoring each item of the tool. They are not intended to cover all relevant issues for all health centres.

Use of the tool provides a score for the state of development of different aspects of health centre systems. The scores may be used as a guide for where improvement efforts might be focussed, but centres should base their priorities on the full range of information available to them and the opportunities they have for improvement in different areas.

References to resources relevant to different clients groups are provided at the end of the tool.

We welcome feedback on the SAT.

Components of the Systems Assessment

Delivery system design<br>This component refers to the extent to which the design of the health centre's infrastructure, staffing profile and allocation of roles and responsibilities, client flow and care processes maximise the potential effectiveness of the centre
Information systems and decision support<br>This component refers to the clinical and other information structures (including structures to support clinical decision making) and processes to support the planning, delivery and coordination of care.
Self-management support<br>This component refers to structures and processes that support clients and families to play a major role in maintaining their health, managing their health problems, and achieving safe and healthy environments.
Links with community, other health services and other services.<br>This component refers to the extent to which the health centre uses external linkages to inform service planning, links clients to outside resources, works out in the community, and contributes to regional planning and resource development.
Organisational influence and integration.<br>This component refers to the use of organisational influence to create and support organisational structures and processes that promote safe, high quality care; and how well all system components are integrated across the centre.

Components	Items for each component
Delivery system design	Team structure and function<br>Clinical leadership<br>Appointments and scheduling<br>Care Planning<br>Systematic approach to follow-up<br>Continuity of care<br>Client access/cultural competence<br>Physical infrastructure, supplies and equipment
Information systems and decision support	Maintenance and use of electronic client lists<br>Evidence based guidelines<br>Specialist-generalist collaborations
Self-management support	Assessment and documentation<br>Self-management education and support, behaviour risk reduction and peer support
Links with community, other health services and other services	Communication and cooperation on governance and operation of the health centre and other community based organisations and programs<br>Linking health centre clients to outside resources<br>Working in the community<br>Communication and cooperation on regional health planning and development of health resources
Organisational influence and integration	Organisational commitment<br>Quality improvement strategies<br>Integration of health system components

Component 1 Delivery system design

1.1 Team structure and function

Elements for discussion	Participants score			
Team approach<br>Is there security and ongoing availability of all the practitioners required	No team approach; practitioners needed for team approach not available	Some efforts to establish a team approach; practitioners needed for team approach sometimes available, but not secure or ongoing	Team approach becoming well established; practitioners needed for team approach usually available, becoming more secure and ongoing	Fully established team approach; secure, ongoing availability of practitioners needed for team approach
Leadership<br>Is it defined and recognised?<br>Does the leader have an appropriate level of formal authority within the practice team?		Team leadership not clearly defined	Team leadership becoming defined and recognised, leader acquiring formal authority	Team leadership clearly defined and recognised, leader has formal authority.
Definition of roles and responsibilities and lines of reporting.<br>Is these defined for all team members?<br>Are these integrated into the delivery system?		Definition of team roles, lines of reporting and integration in system design are fair	Definition of team roles, lines of reporting and integration in system design are good	Definition of team roles, lines of reporting and integration in system design are very good
Communication and cohesion<br>Does this exist within the team?<br>Does the team meet regularly?<br>Are there established processes for effective decision making?		Fair communication and cohesion within the team; team meets irregularly; decision-making is fair	Good communication and cohesion within the team; team meetings becoming regular; decision-making is good	Very good communication and cohesion within the team; team meetings regular; decision-making is very good
Developing team members' skills and roles<br>Is there a strategic approach?		Development of team members' skills and roles is fair	Development of team members' skills and roles is good	Development of team members' skills and roles is very good
Score	0 1 2	3 4 5	6 7 8	9 10 11

1.2 Clinical leadership

Elements for discussion	Participants score											
Clinical leadership<br>Is it fully established and recognised in the area?	No or minimal clinical leadership			Clinical leadership emerging			Clinical leadership becoming established and recognised			Clinical leadership fully established and recognised		
Contribution<br>Does clinical leadership contribute to the centre's vision for high quality care for the client group?				Contribution of clinical leadership to centre's vision for high quality care is fair			Contribution of clinical leadership to centre's vision for high quality care is good			Contribution of clinical leadership to centre's vision for high quality care is very good		
Knowledge about research evidence<br>Does clinical leadership help to ensure that the centre remains knowledgeable about research evidence? Is the evidence interpreted and appropriately applied to the centre's clinical services and population programs?				Contribution of clinical leadership to knowledge and application is fair			Contribution of clinical leadership to knowledge and application is good			Contribution of clinical leadership to knowledge and application is very good		
Score	0	1	2	3	4	5	6	7	8	9	10	11

1.3 Appointments and scheduling

Elements for discussion	Participants score											
Appointment system<br>Is there an established appointment system for this area?<br>Does it have the flexibility to systematically accommodate the needs of the client group including – drop-ins, long or family consultations, clients seeing multiple providers in a single visit as required?	No appointment system			Some appointments made; flexibility is ad hoc			Appointment system becoming established; flexibility becoming systematic			Appointment system fully established; flexibility is systematic		
Specific clinics and /or sessions<br>Are there clinics/sessions with the specialist support available (as appropriate)?<br>Are these clinics/sessions part of routine practice in this area	Specific clinics and/or sessions not used			Specific clinics and/or sessions used in ad hoc way			Specific clinics and/or sessions becoming part of routine practice			Specific clinics and/or sessions part of routine practice		
Planning and scheduling<br>Is it routine practice for the service's community based activities and programs in this area to be planned/scheduled ahead of time?	No or few community based activities			Scheduling of activities/programs is ad hoc			Planning/scheduling of activities/programs becoming routine practice			Planning/scheduling of activities/programs is routine practice		
Score	0	1	2	3	4	5	6	7	8	9	10	11

1.4 Care Planning

Elements for discussion	Participants score											
Routine practice<br>Is care planning for clients part of routine practice?	No or minimal care planning			Care planning is ad hoc			Care planning becoming part of routine practice			Care planning part of routine practice		
Elements of care planning<br>Is it consistent with best practice guidelines?<br>Is it done jointly by providers and clients/families?<br>Includes goal setting/incorporates self management goals and strategies				Some elements included			Most elements included			All elements included		
Score	0	1	2	3	4	5	6	7	8	9	10	11

1.5 Systematic approach to follow-up

Elements for discussion	Participants score											
Electronic flags and reminders<br>Are they used to support client care in this area?<br>Is their use consistent across the clinical area?	No electronic flags/reminders			Flags/reminders sometimes used to support client care			Flags/reminders usually used to support client care			Flags/reminders consistently used to support client care		
Regular services and reviews<br>Are clients followed-up in accordance with best practice?<br>Is this part of routine practice?	No or minimal follow-up of clients			Follow-up of clients for regular reviews is ad hoc			Follow-up of clients for regular reviews is becoming part of routine practice			Follow-up of clients for regular reviews is routine practice		
Abnormal pathology and other test results<br>Is follow-up a systematic part of routine practice?	No or minimal processes for following up abnormal results			Follow-up of abnormal test results is ad hoc			Follow-up of abnormal test results is becoming part of routine practice			Follow-up of abnormal test results is routine practice		
Health centre staff and community knowledge and resources are used to enhance follow-up<br>Does it balance duty of care with client self-management?	No or minimal use of available resources to enhance follow-up			Use of available resources to enhance follow-up is fair			Use of available resources to enhance follow-up is good			Use of available resources to enhance follow-up is very good		
Score	0	1	2	3	4	5	6	7	8	9	10	11

1.6 Continuity of care

Elements for discussion	Participants score			
**Delivery system is designed to enhance continuity of care in this area by having the following elements**<br>Well organised electronic clinical records and clear documentation<br>Scheduled follow-up visits<br>Continuity of provider(s)<br>Team care<br>Case management<br>Shared client records<br>Orientation of health centre staff to processes to enhance continuity of care.	Delivery system is not designed to enhance continuity of care	Delivery system beginning to be designed to enhance continuity of care (some elements in place)	Delivery system quite well designed to enhance continuity of care (most elements in place)	Delivery system very well designed to enhance continuity of care (all or almost all elements in place)
**Communication between hospital(s) and health centre**<br>Is the system effective following discharge of clients in this area?	No or minimal communication between hospital and the health centre post-discharge	Post-discharge communication between hospital and the health centre is on an ad hoc basis only	System for routine post-discharge communication between hospital and the health centre becoming established	System for routine post-discharge communication between hospital and the health centre fully established
Score	0 1 2	3 4 5	6 7 8	9 10 11

1.7 Client access/cultural competence

Elements for discussion	Participants score			
Physical, communication and transport barriers to access<br>Do health centre design and processes address<br>client privacy and confidentiality<br>The use of translators (as required)<br>Transport support for referrals	No or minimal attention given to barriers	Barriers beginning to be addressed but many remain	Barriers addressed quite well but some remain	Barriers addressed very well and few or none remain
Staffing<br>Is there a systematic approach to ensuring that all health centre staff providing care are culturally competent through staff orientation and training?	No or minimal attention given to cultural competence; not included in orientation and training	Level of attention to cultural competence is fair; sometimes included in orientation and training	Level of attention to cultural competence is good; usually included in orientation and training	Level of attention to cultural competence is very good; always included in orientation and training
Gender-related issues<br>Is there a process in place to ensure respect is applied for gender related issues?	No or minimal respect for gender-related issues	Respect for gender-related issues is fair	Respect for gender-related issues is good	Respect for gender-related issues is very good
Indigenous knowledge and AHW experience<br>Is indigenous knowledge and Aboriginal Health Worker experience respected?<br>Does it inform clinical practice and community based activities?	No or minimal respect for Indigenous knowledge or AHW experience	Respect for Indigenous knowledge and AHW experience is fair	Respect for Indigenous knowledge and AHW experience is good	Respect for Indigenous knowledge and AHW experience is very good
Score	0 1 2	3 4 5	6 7 8	9 10 11

1.8 Physical infrastructure

Elements for discussion	Participants score											
Physical infrastructure<br>Is it suitable for provision of care?	Physical infrastructure unsuitable			Physical infrastructure somewhat suitable			Physical infrastructure quite suitable			Physical infrastructure highly suitable		
Supplies of consumables<br>Are they appropriate and available?	Appropriateness and availability of consumables is poor			Appropriateness and availability of consumables is fair			Appropriateness and availability of consumables is good			Appropriateness and availability of consumables is very good		
Equipment<br>Is it appropriate and available?<br>Is it of good quality and very well maintained (e.g .does not need to be shared between or borrowed from other consulting areas due to limited availability or poor maintenance?)	Equipment appropriateness, quality and maintenance is poor			Equipment appropriateness, quality and maintenance are fair			Equipment appropriateness, quality and maintenance are good			Equipment appropriateness, quality and maintenance are very good		
Score	0	1	2	3	4	5	6	7	8	9	10	11

Component 2 Information systems and decision support

2.1 Maintenance and use of electronic client list

Elements for discussion	Participants score											
Electronic list of clients<br>Is one available?<br>Is it regularly reviewed according to an established protocol?<br>Is it up to date, including record of place of residence and Medicare number?	No electronic list			List available but not reviewed and out of date (covers less than 80% of clients, up-to-date residence and Medicare information sometimes recorded)			List available, irregularly reviewed and reasonably up to date (covers 80% or more of clients, up-to-date residence and Medicare information usually recorded)			List available, regularly reviewed and up to date (covers all clients, up-to-date residence and Medicare information always recorded)		
Regular clients<br>Electronic list is routinely used to identify support service planning and delivery? For example, identifying clients for preventive and early detection services according to demographic and risk characteristics.				Use of the list to identify regular clients for planning and delivery is ad hoc			Use of list to identify regular clients for planning and delivery becoming routine			Use of list to identify regular clients for planning and delivery is routine		
Regular clients with specific conditions<br>Electronic list is used to identify to support service planning and delivery? E.g to generate lists of clients for follow-up or regularly scheduled services.				Use of the list to identify regular clients with specific conditions for planning and service delivery is ad hoc			Use of the list to identify regular clients with specific conditions for planning and service delivery becoming routine			Use of the list to identify regular clients with specific conditions for planning and service delivery is routine		
Reaching client groups<br>Are strategies implemented as part of routine practice				Implementation of strategies to reach client groups is ad hoc			Implementation of strategies to reach client groups becoming routine practice			Implementation of strategies to reach client groups is routine practice		
Score	0	1	2	3	4	5	6	7	8	9	10	11

2.2 Evidence based guidelines

Elements for discussion	Participants score											
Evidence-based guidelines and other resources<br>Are they suitable to the service setting?<br>Are they available and accessible electronically?	No or minimal availability or accessibility of electronic evidence-based resources			Availability and accessibility of electronic evidence-based resources is fair			Availability and accessibility of electronic evidence-based resources is good			Availability and accessibility of electronic evidence-based resources is very good		
Evidence-based guidelines and other resources<br>Are they used as part of routine practice	No or minimal use of evidence-based resources			Use of evidence-based resources is ad hoc			Use of evidence-based resources becoming part of routine practice			Use of evidence-based resources is part of routine practice		
Training and /or orientation<br>Is training /orientation to the use of these resources well integrated into in-service training?	No or minimal staff training in use of evidence-based resources			Staff training in use of evidence-based resources is fair			Staff training in use of evidence-based resources is good			Staff training in use of evidence-based resources is very good		
Score	0	1	2	3	4	5	6	7	8	9	10	11

2.3 Specialist and generalist collaborations

Elements for discussion	Participants score											
Specialist – generalist collaboration<br>Is there a strategic approach that results in:<br>• Enhanced decision support for clinical care<br>• Effective generalist-specialist communication about client needs and care<br>• Culturally appropriate care across the spectrum of generalist-specialist care<br>Specialist engagement in the development of community-based programs that promote healthy social and physical environments.	No or minimal specialist-generalist collaboration – i.e. traditional referral only			Specialist-generalist collaboration is fair			Specialist-generalist collaboration is good			Specialist-generalist collaboration is very good		
Score	0	1	2	3	4	5	6	7	8	9	10	11

Component 3 Self-management support

3.1 Assessment and documentation

Elements for discussion	Participants score			
Self-management for clients in this area is supported as a central, strategic part of health care.	No or minimal support for self-management	Fair support for self-management	Good support for self-management	Very good support for self-management
Self-management needs for clients in this area are routinely assessed and documented in a standardised way.	Self-management needs are rarely assessed	Self-management needs sometimes assessed and documented but on an ad hoc basis only	Assessment and documentation of self-management needs becoming routine practice	Assessment and documentation of self-management needs is routine practice
Clients/families in this area are routinely engaged in the assessment and documentation processes.	No or minimal engagement of clients/families in assessment processes	Clients/families engagement in assessment and documentation is ad hoc	Clients/families engagement in assessment and documentation becoming routine practice	Clients/families engagement in assessment and documentation is routine practice
Use of client held records to promote self-management is part of routine practice in this area –<br><br>i.e. tools that are designed to assist clients to adhere to self-management programs and to set goals, track their progress and understand the reasons for health visits.	No or minimal use of client held records	Use of client held records is ad hoc	Use of client held records becoming part of routine practice	Use of client held records is part of routine practice
Score	0 1 2	3 4 5	6 7 8	9 10 11

3.2 Self-management education and support, behavioural risk reduction and peer support

Elements for discussion	Participants score			
Self-management education and support<br>Are routinely provided by staff with recognised training and skills in self-management support?	No or minimal self-management education or support	Some self-management education and support by staff with limited training and skills	Good self-management education and support by staff with relevant training and skills	Very good self-management education and support by staff with relevant training and skills
Involvement of families<br>Are families involved in self-management education and support activities as part of routine practice?	No or minimal engagement of families in education/support activities	Engagement of families in education/ support activities but on an ad hoc basis only	Engagement of families in education/ support activities becoming routine practice	Engagement of families in education/ support activities is routine practice
Behavioural risk reduction<br>Is there a systematic approach to behaviour change interventions? For example, brief intervention for alcohol and tobacco risk reduction?<br>Are brief interventions routinely provided by staff with recognised training and skills in behavioural intervention?	No or minimal provision of behaviour change interventions	Some behavioural interventions provided but by staff with limited relevant training and skills	Behavioural interventions by staff with relevant training and skills becoming part of routine practice	Behavioural interventions by staff with relevant training and skills part of routine practice
Educational resources<br>Are good quality educational resources used for clients and families to support behavioural risk reduction self-management?<br>Is this part of routine practice?	No or minimal use of resources to support self-management	Some use of resources to support self-management	Use of resources to support self-management becoming routine practice	Use of resources to support self-management is routine practice
Community peer support<br>Is promotion and support for programs and activities a central, strategic part of health care?	No or minimal promotion or support for peer support	Promotion and support for peer support is ad hoc	Promotion and support for peer support is becoming a central, strategic part of care	Promotion and support for peer support is a central, strategic part of care
Score	0 1 2	3 4 5	6 7 8	9 10 11

Component 4 Links with community, other health services and resources

4.1 Communication and cooperation on governance and operation of the health centre and other community based organisations and programs

Elements for discussion	Participants score										
Community input to health centre governance<br>Are there well-functioning arrangements?	No community input to governance			Community input to governance is fair			Community input to governance is good			Community input into governance is very good	
Involvement of service population<br>Is there a systematic approach to in service planning and feedback?<br>Does it include input through an annual general meeting and reference groups/committees?<br>Does it have formal mechanisms for dissemination of health service performance information?	No service population involvement in planning and feedback			Service population involvement in planning and feedback is ad hoc.			Service population involvement in planning and feedback is becoming systematic			Service population involvement in planning and feedback is systematic	
Client satisfaction with the health centre's services<br>Are they systematically and routinely assessed?	Client satisfaction never or rarely assessed			Assessment of client satisfaction is ad hoc			Assessment of client satisfaction is becoming systematic and routine			Assessment of client satisfaction is systematic and routine	
Formal agreements between the health centre and mainstream primary care services (including Divisions of Primary Care) and other health and community services relevant to this area<br>Are agreements in place?<br>Do they involve good communication and ongoing, strategic activities?	No formal agreements with other services			Formal agreements with other services with fair communication and levels of activity			Formal agreements with other services with good communication and levels of activity			Formal agreements with other services with very good communication and levels of activity	
Score	0	1	2	3	4	5	6	7	8	9	10	11

4.1 continued….

Elements for discussion	Participants score											
Partnership with relevant community groups<br>Are there well-functioning arrangements for the health centre to work in?<br>E.g. municipal councils, schools, women's centres, resource centres, art centres, child care centres, sport and recreation groups, cultural programs.<br>Does this help to ensure community programs have a positive health impact?	No or poor partnerships with community groups			Partnerships with community groups are fair			Partnerships with community groups are good			Partnerships with community groups are very good		
Health orientation<br>Do community, social, education and other programs and organisations have a strong health orientation?	Health orientation of community programs is weak			Health orientation of community programs is fair			Health orientation of community programs is good			Health orientation of community programs is very good		
Score	0	1	2	3	4	5	6	7	8	9	10	11

4.2 Linking health centre clients to outside resources

Elements for discussion	Participants score			
There are systematic arrangements in place to link individual clients in this area to outside health and health-related resources.	No or minimal arrangements for linking clients to outside resources	Arrangements for linking clients to outside resources ad hoc	Arrangements for linking clients to outside resources becoming systematic	Arrangements for linking clients to outside resources are systematic
The resource directory that supports systematic arrangements is comprehensive, regularly updated, is easily accessible and widely used by staff.	No resource directory –	Resource directory – comprehensiveness, updating accessibility and use are fair	Resource directory – comprehensiveness, updating accessibility and use are good	Resource directory – comprehensiveness, updating accessibility and use are very good
Linkage arrangements relating to these resources are well integrated into staff orientation and in-service training programs.	No or minimal integration of linkage arrangements in staff orientation or training	Integration of linkage arrangements in staff orientation or training is fair	Integration of linkage arrangements in staff orientation or training is good	Integration of linkage arrangements in staff orientation or training is very good
Score	0 1 2	3 4 5	6 7 8	9 10 11

4.3 Working out in the community

Elements for discussion	Participants score											
Staff engagement<br>Are staff engaged in community health promotion/development activities?<br>e.g in pre-schools and schools; men's, women's and youth groups; community centres; community stores.	No or minimal staff engagement in community health promotion/ development			Level of staff engagement in community health promotion/development is fair			Level of staff engagement in community health promotion/development is good			Level of staff engagement in community health promotion/development is very good		
Design of community activities<br>Are community activities well-designed?<br>Do they meet identified needs of different groups?				Design of community activities is fair			Design of community activities is good			Design of community activities is very good		
Integration<br>Are community activities fully integrated into the centre's programs?				Integration of community activities into centre's programs is fair			Integration of community activities into centre's programs is good			Integration of community activities into centre's programs is very good		
Score	0	1	2	3	4	5	6	7	8	9	10	11

4.4 Communication and cooperation on regional health planning and development of health resources

Elements for discussion	Participants score			
Regional planning<br>Are health centre staff actively engaged in and promote regional planning?	No or minimal engagement in regional planning	Level of engagement in regional planning is fair	Level of engagement in regional planning is good	Level of engagement in regional planning is very good
Health resources<br>Do health centre staff actively contribute to the development and promotion of standard resources for health services that have region-wide relevance in this area?	No or minimal contribution to the development of resources	Contribution to the development of resources is fair	Contribution to the development of resources is good	Contribution to the development of resources is very good
Local community plans<br>Are plans systematically used to inform regional planning processes and allocation of resources?	No or minimal use of community plans	Use of community plans is ad hoc	Use of community plans is becoming systematic	Use of community plans is systematic.
Score	0 1 2	3 4 5	6 7 8	9 10 11

Component 5 Organisational influence and integration

5.1 Organisational commitment

Elements for discussion	Participants score											
Strategic and business plans<br>Do they reflect commitment to this client group<br>i.e. vision statement, policies, financing, staffing, strategies?	No plans; little or no interest in a plan			Plans in place; level of commitment is fair			Plans in place; level of commitment is good			Plans in place; level of commitment is very good		
Funding<br>Is there specific funding for this area that is at an adequate level and long-term?	No specific funding			Specific funding, level is fair and/or short term			Specific funding, level is good and/or medium term			Specific funding, level is very good and/or long term		
Staffing<br>Do staffing levels meet the established need?<br>Are all the relevant roles defined and these roles reflected in job descriptions?	Minimal staffing; no specific roles			Level of staffing is fair; some roles defined			Level of staffing is good; most roles defined and reflected in job descriptions			Level of staffing is very good; all roles defined and reflected in job descriptions		
Staff relationships and morale<br>Are there good relationships and regular, clear communication among staff?<br>Where is morale high?<br>Is there is a feeling among line staff that senior staff understand their work and needs?	Poor relationships and little or no communication<br>Morale is low			Relationships and communication are fair<br>Morale is fair			Relationships and communication are good<br>Morale is good			Relationships and communication are very good<br>Morale is very good		
Training<br>What is the range of training and in-service opportunities for staff working in this area?	Range of training and in-service opportunities is poor			Range of training and in-service opportunities is fair			Range of training and in-service opportunities is good			Range of training and in-service opportunities is very good		
Service delivery strategies<br>Is there a range of service delivery strategies in this area across individual clinical, group and population based activities (as appropriate)	Range of service delivery strategies is poor			Range of service delivery strategies is fair			Range of service delivery strategies is good			Range of service delivery strategies is very good		
Score	0	1	2	3	4	5	6	7	8	9	10	11

5.2 Quality improvement strategies

Elements for discussion	Participants score											
Senior staff support for quality improvement<br>Do senior staff support quality improvement?<br>Is it resourced?<br>Is staff training provided?<br>Is participation encouraged?<br>Do staff have authority to make improvements?<br>Is effectiveness evaluated?	No or minimal senior staff support for quality improvement			Limited senior staff support for quality improvement			Senior staff support quality improvement but not fully or consistently			Quality improvement fully and consistently supported by senior staff		
Quality improvement processes<br>Are there systematic processes in place?<br>Are they used consistently?<br>e.g. cyclical processes of evidence-based assessment of health centre performance using good quality data, review and planning involving the whole team, and service improvement.	No or minimal quality improvement processes			Ad hoc quality improvement processes			Systematic quality improvement processes but not used consistently			Systematic quality improvement processes used consistently		
Health centre performance reporting<br>Is the electronic client information system routinely used in this area?<br>e.g. including profiles and needs of client groups, care delivery and client outcomes	No electronic client information system			Use of the system for reporting on centre performance is ad hoc			Use of the system for reporting on centre performance becoming routine			Use of the system for reporting on centre performance is routine		
Processes for dealing with errors and problems<br>Are systematic processes in place for dealing with errors or problems with care delivery?<br>Do they include routine identification, examination of root causes and follow through appropriate action and regular review?	No or minimal processes for dealing with errors or problems			Processes for dealing with errors or problems are ad hoc			Processes for dealing with errors becoming systematic			Processes for dealing with errors systematic		
Score	0	1	2	3	4	5	6	7	8	9	10	11

5.3 Integration of health system components

Elements for discussion	Participants score											
Integration<br>There is clear recognition of the need for and importance of integration across the health centre.<br>How well the information system supports clinical decision making (by making guidelines accessible) or self-management (by allowing recording of client goals)<br>How well the funding and human resources arrangements support team care<br>How well work within and outside the health centre complement each other<br>How well staff training supports continuity of care.<br><br>This is reflected in all documents/processes/activities including:<br>• Business plan<br>• Policy statements<br>• Financing arrangements<br>• Information system<br>• Regulation/legislation<br>• Deployment of human resources<br>• Leadership and advocacy roles<br>• Care processes<br>• Education and in-service programs<br>• Work outside the health centre<br>• Partnership arrangements	No or minimal integration			Fair level of integration			Good level of integration			Very good level of integration		
Score	0	1	2	3	4	5	6	7	8	9	10	11
