# Supplementary material for: Leveraging quality improvement through use of the Systems Assessment Tool in Indigenous primary health care services: a mixed methods study
Source: BMC Health Serv Res. 2016 Oct 18;16:583. doi: 10.1186/s12913-016-1810-y (PMC5070177; doi:10.1186/s12913-016-1810-y)
Supplement: Additional file 2: — Facilitator’s Guide to Systems Assessment Tool, Version 1.0. (PDF 421 kb) [file 12913_2016_1810_MOESM2_ESM.pdf]

Facilitator’s guide to the systems assessment tool

Version 1.0

September 2012

One21seventy
National Centre for Quality Improvement in Indigenous Primary Health Care

menzies
school of health research

© Menzies School of Health Research 2012

This material is copyright. Apart from use permitted under the *Copyright Act 1968* (Cwlth), all other rights are reserved. No part of this material may be reproduced, by any process, without prior written permission. Parts of this material are available to registered users for download from the One21seventy web-based information system, www.one21seventy.org.au. Requests and enquiries concerning reproduction and rights should be addressed to: Menzies School of Health Research, Darwin Northern Territory, Australia.

First published 20012

Acknowledgments

We would like to acknowledge and thank all the members who participated in the working group:

Jenny Brands, Michel Burgum, Kerry Copley, Cynthia Croft, Sue Ferguson-Hill, Jenny Hains, Cath Kennedy, Ru Kwedza, Alison Laycock, Diana Mosca, Estrella Munzo, Louise Patel, Leticia Robertson.

Contents

Notes for the facilitator iii

About systems assessment....... 1

The One21seventy systems assessment tool 2

Facilitating a systems assessment 5

History of the One21seventy Systems Assessment Tool (SAT) 10

Version control

Version	Release date	Description
1.0	September 2012	Launch

Notes for the facilitator

1. It is recommended that you have attended the foundation training and have read and understood section 6 and 10 of the Training Manual for the One21seventy cycle.
2. There is an expectation that the facilitator will have a sound level of health literacy to be able to interpret the concepts and guide discussion by using the elements for discussion for each component item.
3. Although not always necessary services have found it more productive and quicker to complete the Systems Assessment by including participants who have attended the foundation training.
4. The Systems Assessment tool concepts have not been placed in order of importance or priority and it is up to you and your team to determine the order you want to progress through them.
5. Some of the score descriptors may not appear in the 0-2 columns this means that if you answer no to the first question the following questions for that item are not applicable and a score of 0 is used.
6. Additional help is available if you require, by calling the One21seventy helpdesk at any time during the week between 0830-1630hrs Monday to Friday Brisbane time.

Resources to use for systems assessment discussion

- Improving the quality of primary health care: a training manual for the One21seventy cycle.
- Facilitator's guide to the systems assessment
- Systems Assessment Tool (SAT)
- Systems assessment scoring form
- One21seventy help desk: 1800 082 474 or one21seventy@menzies.edu.au.

About systems assessment

This facilitator's guide should be used in conjunction with Section 6 of *Improving the quality of primary health care: A training* manual for the One21seventy CQI cycle.

What is a system?

A system can be defined as a set of interacting parts, which form an integrated whole. Systems can be simple or complex. For example, a wheelbarrow is a simple system, with a few parts mechanically bound together. Human society is a complex system, with numerous parts linked together through sets of relationships. If one part of the system is removed or changed, the nature of the system is changed. The same can be said of organisational systems.

It is widely accepted that quality of care can be seriously limited by poor organisational systems. Improving organisational systems is therefore very important to improving the quality of clinical care.

What is a systems assessment?

A systems assessment is a process through which the strengths and weaknesses of a system can be identified. The purpose of a systems assessment is generally to determine how the functioning of a system might be improved, with the rationale that better functioning systems are more effective in producing the results they are designed to achieve.

Growing evidence from around the world suggests that patients do better when they receive effective treatments, self-management support, and regular follow-up. Organised systems of care, not just individual health care workers, are essential in producing positive outcomes.

The One21seventy systems assessment tool

What is the One21seventy Systems Assessment Tool?

The One21seventy Systems Assessment Tool (SAT) has been developed to allow local health centres to undertake a structured assessment of the strengths and weaknesses of their systems to support client care. It is based on international and Australian evidence about how health centre systems can achieve high quality care (see Appendix 6A). The tool allows systems assessments to be carried out in a comparable way at different times and in different places, and in a way that covers the key components of health centre systems.

The SAT produces a set of scores and score justifications for a local health centre that are reported along with the results of their clinical audits. Together these reports can be used to:

- identify strengths and weaknesses of the health centre system
- identify priorities for improvement
- develop strategies to address those priorities
- develop action plans for implementation of those strategies.

What are the origins of the One21seventy SAT?

The One21seventy SAT was developed by the Menzies School of Health Research as part of the Audit and Best Practice in Chronic Disease (ABCD) Project. It evolved from the Chronic Care Model and the associated Assessment of Chronic Illness Care (ACIC) tool developed in the United States in the 1990s[1], and from the World Health Organization's (WHO) Innovative Care for Chronic Conditions (ICCC) Framework[2]. (For links to original sources about the Chronic Care Model, the ACIC scale and the ICCC Framework, go to the www.one21seventy.org.au, and select the 'CQI and Indigenous health' menu available from the home page before logging in.)

A more detailed description of the origins of the SAT is included in Appendix 6A at the end of this section.

[1] Bonomi AE, Wagner EH, Glasgow RE, VonKorff M: Assessment of chronic illness care (ACIC): a practical tool to measure quality improvement. Health Serv Res 2002, 37:791-820.

[2] WHO: *Innovative care for chronic conditions: building blocks for action.* Geneva: WHO; 2002.

What are the components of the One21seventy SAT?

The first step in improving systems is to understand the components of health systems that can promote good quality of care. International experience has identified five key components of health systems to be effective across primary health care in improving the quality of care of clients with chronic illness.

- Delivery system design
- Information systems and decision support
- Self-management support
- Links with the community, other health services and other services and resources
- Organisational influence and integration

These five components are incorporated into the One21seventy SAT. Within each component are a number of items as described in Table 6.1. Each item is scored separately. Items incorporate various elements to prompt discussion; elements can be scored and averaged to determine an item score.

The One21seventy SAT requires different scores and justifications to be made for each audit tool (chronic disease, maternal health, etc.). This is because the quality of systems to support different aspects of care may differ substantially.

How is the systems assessment carried out at the local health centre?

The systems assessment can itself be an important change process because it requires local health centre staff and managers to discuss and come to a consensus about how well their systems are working. This may be the only time that this occurs on a whole-of-health-centre basis.

Ideally, a systems assessment will be done with an external facilitator who conducts the systems assessment with all the local health centre staff and managers. This approach enables a sharing of perspectives from all staff about how the health centre systems function. A good facilitator helps to ensure that all members of the group are able to participate and share their views. Different staff may have very different perspectives on how the systems function. Having staff share their perspectives contributes to a wider shared understanding of the strengths and weaknesses in the system. It is worth trying to find a time when everyone can participate in the systems assessment.

What happens to the systems assessment data once collected?

Data input

The systems assessment scores and justifications are entered into the One21seventy web-based information system (www.one21seventy.org.au) in the 'Input Data' section.

Data input can be done by:

- A staff member or designated scribe who is able to accurately reflect the scoring and justifications of the systems assessment.
- The facilitator of the systems assessment.

Generating reports

The relevant SAT results can be viewed on screen in the One21seventy web-based information system, and are presented at the end of the downloadable report for each clinical audit tool. When an audit report is generated, the web-based information system will look for a systems assessment completed within three months before or after the date of that audit, and include that systems assessment report within the audit report.

To view systems assessment data on screen, go to the 'Reports' section of the One21seventy web-based information system (www.one21seventy.org.au).

> Keep in mind that data entry of the One21seventy SAT is an opportunity to build capacity and ownership of data by the local health centre.

Facilitating a systems assessment

These step-by-step instructions are to assist those who facilitate systems assessment for a local health centre. These facilitators may be a regional CQI coordinator, local CQI facilitator or an external facilitator.

Step 1: Preparing for a systems assessment

A few simple preparations will help facilitators get the most out of the systems assessment process for the local health centre staff and managers.

- ☑ Review the SAT and scoring form—download the current version from the One21seventy web-based information system (www.one21seventy.org.au), 'Resources' section.
- ☑ Arrange and subsequently confirm the availability of health centre staff and the time and place for the systems assessment.
- ☑ Find out who will be attending, and what their roles at the health centre are.
- ☑ Check the suitability of the room, availability of any resources needed (such as a data projector, butcher's paper, or a whiteboard) and make arrangements for refreshments to be available.
- ☑ Check how much time has been allocated for the session and plan the session accordingly.
- ☑ If possible, arrange for a senior manager or even a Board member to introduce the session. This will convey the importance that the health centre places on the process.

Identify a scribe

If possible, have a designated scribe identified prior to the systems assessment session. The scribe's role is to write down the agreed scores and justifications as to why the score has been allocated by the group.

The scribe needs to be someone trusted by the group. It is a job that may be difficult for anyone who is not experienced in summarising information from what may be a robust discussion. There are techniques for making sure that the group is happy with what has been recorded, such as:

- Reading back to the group what has been written down and checking with the group it is correct: "Does that capture what you've agreed?" or "Have I got that right?"
- Writing the scores and justifications for the scores onto a whiteboard or directly into the scoring form projected onto a screen by a data projector.
- Another alternative is for the facilitator to be the scribe, though this can be difficult if there is a lot of lively debate.

Step 2: Decide how the systems assessment will be scored

There are two different approaches to scoring a One21seventy systems assessment at a local health centre. It is preferable to determine which method will be used prior to the systems assessment session, so that there is no confusion about the approach. Facilitators should discuss the approach to be used with the local health centre key contact or managers.

How are the SAT items scored?

The One21seventy SAT measures the degree of support provided through the systems related to each item in the five components, with a score ranging from 0–11. The higher the score, the better the system.

The scores are sub-divided into four categories, defined as:

- limited or no support (a score of 0–2)
- basic support (a score of 3–5)
- good support (a score of 6–8)
- fully developed support (a score of 9–11).

The system elements that make up each item are listed under the item heading.

The One21seventy SAT includes brief descriptors to help the health centre staff decide on the category of support their existing systems provide, and agree a score within the level they think best represents the systems in their centre. The facilitator helps the group reach a consensus score.

The scoring for each component can be done in one of two ways:

- Approach One: Calculate the average of scores for all of the items in the component.
- Approach Two: Come to a consensus on an overall score after discussion about the scores and relative importance of each item to the overall component score.

There are pros and cons to each of the approaches. Facilitators should take into account which approach is most suited to their own style and to the staff and circumstances of the health centre with which they are working.

Approach One: Calculate the average score

Component scores are calculated by adding together the item scores and dividing the total by the number of items.

For example, in Component 3 ('Service Delivery System) there are six items:

- Physical layout, supplies and equipment
- Cultural competence of staff and client access to health
- Team structure and function
- Appointments and scheduling
- Care planning and continuity of care
- Systematic approach to follow-up

During the systems assessment, each of the items will be given a score between 0 and 11 (for example, these scores may be 3, 4, 6, 4, 7, 2, 4 and 5, respectively).

The calculation of the component score is done by adding the score for all eight items together (for example 3+4+6+4+7+2+4+5 = 35). This total is then divided by the number of items (8) in this component (for example, 35/8 = 4.375, or 4.4 if the number is rounded to one decimal place).

The advantage of this approach is that it is an easy mathematical way of getting an overall score for each of the components. However, there are a number of issues to consider in using this overall average score approach:

- For the average of the item scores to be a good reflection of the overall component score requires that the scores of 0 to 11 are evenly spaced measures of the state of development of each particular item that makes up the component score.
- It also requires that each of the items is of equal relative importance in the assessment of the overall component.

These conditions may not necessarily be met, so the average score for the items in the component is not necessarily a good overall reflection of the overall component.

Approach Two: Come to a consensus on an overall score

This approach involves a facilitated discussion to help the group reach agreement on an overall score, after discussion about the scores and relative importance of each item to the overall component score.

Approach Two requires skilled facilitation and may be more challenging and time consuming. However, the benefit of this approach is that the dialogue may result in a better shared understanding among health centre staff about existing health centre systems, their relative importance to the quality of care provided, their relative state of development, and potential for improvement.

The challenge is that different people in the group may have different views on the relative importance of each item to the overall component score. Some may have more forceful personalities and may exert undue influence on the outcome of the discussion. Facilitators need to ensure that the consensus score is a fair reflection of the views of all people in the group. (See Section 10, 'Facilitation', for more information on facilitation techniques.)

Achieving a meaningful score

It is common that when a local health centre begins using the One21seventy SAT, its agreed scores may be below '5' on some (or all) components. After all, if all health centre systems worked perfectly and everyone provided optimal care, there would be no need for quality improvement programs.

It is also common for local health centre staff to initially believe they are providing better care than they actually are. As local health centres continue to use the SAT, staff and managers will become more familiar with what an effective health centre system involves.

Health centres SAT scores may decline initially, even though they have made improvements. This is most likely the result of their improved understanding of what a good system of care looks like. Over time, as the understanding of good care increases, and effective changes are implemented, the SAT score should start to rise again.

An important aspect of facilitating the SAT is encouraging the health centre staff and managers to arrive at a consensus decision on system development and to provide a score based on the information discussed in reaching that consensus. (See Section 10, 'Facilitation', for information on effective facilitation.)

Once a consensus score for an item has been reached, the score is entered by the designated scribe onto the scoring form along with a justification that explains why that score was given.

It is important to record score justifications, so that the meaning is clear at the time of doing the systems assessment and when the SAT results are reviewed at a later date. Clear and meaningful recording enhances the value of the SAT for the purpose of improving systems.

A useful way to ensure score justifications are meaningful is to discuss and then record the structures and processes the centre has or doesn't yet have for each SAT item. The score justification recorded should reflect the score decided by the participating staff.

Step 3: Facilitating the systems assessment session

Introductions and explanations

Ideally, a senior manager or even a health centre board member will be at the session and introduce the session and the facilitator.

The facilitator's role in the session begins with introductions:

- Carry out introductions.
- Set out the purpose of the session.

Briefly explain:

- The definition of a system and why organisational systems are important in health care.

> It is courtesy to acknowledge the traditional owners of the area in an introduction, but spend some time practicing the pronunciation of the name/s so as not to cause offence

- What a systems assessment is and why it is useful to a health centre.
- What the One21seventy Systems Assessment Tool (SAT) is and how it was developed.

> While working through the SAT items, ask the group what would be needed to get a better score. Record this on a whiteboard or butcher's paper. These ideas can be used in conjunction with audit reports for action planning

- The components and items of a One21seventy Systems Assessment Tool score sheet. Showing these from a data projector or handing some copies round can help health centre staff understand how the assessment is done.
- How the scores and justifications will be determined. Explain everyone will have the opportunity to contribute their perspective before the group decides on a consensus score.

> Health centres might set themselves the challenge of suggesting ways to improve in any area where they score less than 5

- What happens to the systems assessment data after the session?
- How the results are reported and how that information can be used.
- Establish the 'ground rules' for the session and the role of the facilitator. (See Section 10, 'Facilitation', for more information on facilitation techniques.)
- If necessary, get the group to confirm the scribe and show him/her the layout of the score sheet and what to do. Ask the scribe to begin by completing the names and positions of participating staff. (This may have been done prior to beginning the session.)

Working through the components and items

- Start with the first component item ('Delivery System Design'). Work with the group in order for them to decide which of the score descriptors best reflects their current situation and help them to reach a consensus.
- Ask the scribe to record the score in the appropriate box on the scoring form.
- Summarise back to the group what is to be recorded about the justification for that score, and get consensus that it is accurate.
- Continue to move through the component items of the SAT.
- At the completion of the SAT, inform the group that the information recorded by the scribe will be entered onto the One21seventy web-based information system and that a full report will be sent back to the key contact person at the local health centre.
- Briefly revisit the stages and actions of the One21seventy CQI cycle which follow the completion of the systems assessment.

History of the One21seventy Systems Assessment Tool (SAT)

The One21seventy Systems Assessment Tool (SAT) has evolved from the Chronic Care Model and the associated Assessment of Chronic Illness Care (ACIC) tool developed in the United States, and from the WHO Innovative Care for Chronic Conditions (ICCC) Framework.

It was originally designed for assessing systems for chronic disease care, and then adapted for use in maternal and child health.

The Chronic Care Model and the ACIC scale

In the late 1990s, the McColl Institute in the United States developed a tool for assessment of organisational systems relevant to chronic illness care: The Assessment of Chronic Illness Care (ACIC) scale[1]. The ACIC scale addresses the basic elements for improving chronic illness care at community, organisation, practice and client levels.

The ACIC scale was developed out earlier work by the same group: the Chronic Care Model. The Chronic Care Model identified six system elements of a primary health care organisation that should support high-quality chronic illness care.

The Chronic Care Model has been the subject of wide international attention and formed the basis of a World Health Organisation (WHO) framework for Innovative Care of Chronic Conditions (ICCC)[2].

The ICCC framework includes attention to the broader policy environment: legislation, financial arrangements for service provision, governance, relationships and collaborations, governmental support, and workforce education.

For more information and links to original sources about the Chronic Care Model, the ACIC scale and the ICCC Framework, go to www.one21seventy.org.au, and select the 'CQI and Indigenous health' menu available from the home page (before logging in).

Development of the One21seventy Systems Assessment Tool

The One21seventy Systems Assessment Tool was developed as part of the ABCD project (See Section 2, 'Introduction to One21seventy'), drawing on the McColl Institute's ACIC model and the WHO's ICCC Framework. These were found to be highly relevant to the Australian Indigenous primary care environment[3].An adapted version of the ACIC tool was used in the original ABCD Project. Over time, the SAT was adapted and refined to maximise its suitability to the local context and for use with clinical audits across a range of areas.

---

[1] Bonomi AE, Wagner EH, Glasgow RE, VonKorff M: Assessment of chronic illness care (ACIC): a practical tool to measure quality improvement. Health Serv Res 2002, 37:791-820.

[2] WHO: *Innovative care for chronic conditions: building blocks for action.* Geneva: WHO; 2002.

[3] Si D, Bailie R, Connors C, Dowden M, Stewart A, Robinson G, Cunningham J, Weeramanthri T: Assessing health centre systems for guiding improvement in diabetes care. *BMC Health Services Res* 2005, 5:56
