# Supplementary material for: Leveraging quality improvement through use of the Systems Assessment Tool in Indigenous primary health care services: a mixed methods study
Source: BMC Health Serv Res. 2016 Oct 18;16:583. doi: 10.1186/s12913-016-1810-y (PMC5070177; doi:10.1186/s12913-016-1810-y)
Supplement: Additional file 3: — Systems Assessment Scoring Form, Version 2.0. (PDF 74 kb) [file 12913_2016_1810_MOESM3_ESM.pdf]

Systems assessment scoring form

Options for scoring

1. A single Systems Assessment for whole of the service

If your health service has decided to complete a single Systems Assessment you will be required to answer '1-Yes' to all service delivery areas listed (in figure 1). Copy all scores and justifications into data entry text boxes for each numbered item on the web site. This will ensure your System Assessment results will appear in each of the clinical audit reports generated.

2. A single service delivery area Systems Assessment

A separate scoring form will need to be completed for a service delivery area if the Systems Assessment is to be conducted by an individual team. In this case only answer '1-Yes' to the service delivery area selected (figure 1).

Health centre code:

Date:

Name of persons participating in the systems assessment:

Service delivery areas covered by this Systems Assessment

Indicate the areas that will be covered in this systems assessment.

Figure 1: Service delivery areas

Child health	1-Yes	0-No
Maternal health	1-Yes	0-No
Mental health	1-Yes	0-No
Preventive services	1-Yes	0-No
RHD/ ARF	1-Yes	0-No
Vascular & Metabolic	1-Yes	0-No

Using the scoring form

Provide an agreed score and a sentence explaining the justification for the score your health service decides upon for each item.

The overall numerical score and justifications are entered into the One21seventy database. A maximum of 500 characters can be entered for each justification.

Contact us

For more information or to provide feedback on the Systems Assessment please contact One21seventy Phone: 1800 082 474 or email one21seventy@menzies.edu.au.

Component 1 Delivery system design

1.1 Team structure and function

Element for discussion	Score	Justification for overall score (500 characters)
Team approach		
Team leadership		
Definition of roles and responsibilities		
Communication and cohesion		
Developing team members skills and rolls		
**Overall score either by average or consensus approach.**		

1.2 Clinical leadership

Element for discussion	Score	Justification for overall score
Clinical leadership		
Contribution		
Knowledge about research evidence		
**Overall score either by average or consensus approach.**		

1.3 Appointments and scheduling

Element for discussion	Score	Justification for overall score
Appointment system		
Specific clinics and / or sessions		
Planning and scheduling		
**Overall score either by average or consensus approach.**		

1.4 Care Planning

Element for discussion	Score	Justification for overall score
Routine practice		
Elements of care planning		
**Overall score either by average or consensus approach.**		

1.5 Systematic approach to follow-up

Element for discussion	Score	Justification for overall score
Electronic flags and reminders		
Regular services and reviews		
Abnormal pathology and other test results		
Health centre staff and community knowledge and resources are used to enhance follow-up		
**Overall score either by average or consensus approach.**		

1.6 Continuity of care

Element for discussion	Score	Justification for overall score
Delivery system designed to enhance continuity of care		
Communication between hospital(s) and health centre		
**Overall score either by average or consensus approach.**		

1.7 Client access/cultural competence

Element for discussion	Score	Justification for overall score
Physical, communication and transport barriers to access		
Staffing		
Gender-related issues		
Indigenous knowledge and AWH experience		
**Overall score either by average or consensus approach.**		

1.8 Physical infrastructure

Element for discussion	Score	Justification for overall score
Physical infrastructure		
Supplies of consumables		
Equipment		
**Overall score either by average or consensus approach.**		

Component 2 Information systems and decision support

2.1 Maintenance and use of electronic client list

Element for discussion	Score	Justification for overall score
Electronic list of clients		
Regular clients		
Regular clients with specific conditions		
Reaching client groups		
**Overall score either by average or consensus approach.**		

2.2 Evidence based guidelines

Element for discussion	Score	Justification for overall score
Evidence-based guidelines and other resources		
Evidence-based guidelines and other resources are used as part of routine practice		
Training and /or orientation		
**Overall score either by average or consensus approach.**		

2.3 Specialist and generalist collaborations

Element for discussion	Score	Justification for overall score
Specialist – generalist collaboration		
**Overall score either by average or consensus approach.**		

Component 3 Self-management support

3.1 Assessment and documentation

Element for discussion	Score	Justification for overall score
Self-management for clients is strategically supported		
Self-management is routinely assessed and documented		
Clients/families are routinely engaged in assessment and documentation		
Use of client hand held records		
**Overall score either by average or consensus approach.**		

3.2 Self-management education and support, behavioural risk reduction and peer support

Element for discussion	Score	Justification for overall score
Self-management education and support		
Involvement of families		
Behavioural risk reduction		
Educational resources		
Community peer support		
**Overall score either by average or consensus approach.**		

Component 4 Links with community, other health services and resources

4.1 Communication and cooperation on governance and operation of the health centre and other community based organisations and programs

Element for discussion	Score	Justification for overall score
Community input to health centre governance		
Involvement of service population		
Client satisfaction with the health centre's services		
Formal agreements between the health centre and mainstream primary care services		
Partnership with relevant community groups		
Health orientation		
**Overall score either by average or consensus approach.**		

4.2 Linking health centre clients to outside resources

Element for discussion	Score	Justification for overall score
Systematic arrangements in place to link clients to outside health resources		
Resource directory		
Linking arrangements is integrated into staff orientation		
**Overall score either by average or consensus approach.**		

4.3 Working out in the community

**Element for discussion**	**Score**	**Justification for overall score**
Staff engagement		
Design of community activities		
Integration		
**Overall score either by average or consensus approach.**		

4.4 Communication and cooperation on regional health planning and development of health resources

**Element for discussion**	**Score**	**Justification for overall score**
Regional planning		
Health resources		
Local community plans		
**Overall score either by average or consensus approach.**		

Component 5 Information systems and decision support

5.1 Organisational commitment

Element for discussion	Score	Justification for overall score
Strategic and business plans		
Funding		
Staffing		
Staff relationships and morale		
Training		
Service delivery strategies		
**Overall score either by average or consensus approach.**		

5.2 Quality improvement strategies

Element for discussion	Score	Justification for overall score
Senior staff support for quality improvement		
Quality improvement processes		
Health centre performance reporting		
Processes for dealing with errors and problems		
**Overall score either by average or consensus approach.**		

5.3 Integration of health system components

Element for discussion	Score	Justification for overall score
Integration		
**Overall score either by average or consensus approach.**		
